# Modeling the parameters for plasmodesmal sugar filtering in active symplasmic phloem loaders

**DOI:** 10.3389/fpls.2013.00207

**Published:** 2013-06-19

**Authors:** Johannes Liesche, Alexander Schulz

**Affiliations:** Department of Plant and Environmental Sciences, University of CopenhagenCopenhagen, Denmark

**Keywords:** phloem loading, plasmodesmata, polymer trap, hindered diffusion, carbon allocation

## Abstract

Plasmodesmata (PD) play a key role in loading of sugars into the phloem. In plant species that employ the so-called active symplasmic loading strategy, sucrose that diffuses into their unique intermediary cells (ICs) is converted into sugar oligomers. According to the prevalent hypothesis, the oligomers are too large to pass back through PD on the bundle sheath side, but can pass on into the sieve element to be transported in the phloem. Here, we investigate if the PD at the bundle sheath-IC interface can indeed fulfill the function of blocking transport of sugar oligomers while still enabling efficient diffusion of sucrose. Hindrance factors are derived via theoretical modeling for different PD substructure configurations: sub-nano channels, slit, and hydrogel. The results suggest that a strong discrimination could only be realized when the PD opening is almost as small as the sugar oligomers. In order to find model parameters that match the *in vivo* situation, we measured the effective diffusion coefficient across the interface in question in *Cucurbita pepo* with 3D-photoactivation microscopy. Calculations indicate that a PD substructure of several sub-nano channels with a radius around 7 Å, a 10.4 Å-wide slit or a hydrogel with 49% polymer fraction would be compatible with the effective diffusion coefficient. If these configurations can accommodate sufficient flux of sucrose into the IC, while blocking raffinose and stachyose movement was assessed using literature data. While the slit-configuration would efficiently prevent the sugar oligomers from “leaking” from the IC, none of the configurations could enable a diffusion-driven sucrose flux that matches the reported rates at a physiologically relevant concentration potential. The presented data provides a first insight on how the substructure of PD could enable selective transport, but indicates that additional factors are involved in efficient phloem loading in active symplasmic loading species.

## Introduction

An essential step for the distribution of carbohydrates throughout the whole plant is the loading of carbohydrates into the phloem in source organs. A number of herbaceous angiosperms, as e.g., the Cucurbits, preferentially transport the sugar oligomers raffinose and stachyose in the phloem, in contrast to the majority of plants where sucrose is transported exclusively (Rennie and Turgeon, 2009; Davidson et al., 2011). They are therefore referred to as raffinose family oligosaccharide (RFO)-transporting plants.

According to our current understanding of phloem transport in these species, the conversion of sucrose into RFOs is of key importance for the loading of sugars into the phloem (Rennie and Turgeon, 2009; Liesche and Schulz, 2013). RFO-transporting plants are also characterized by an abundance of plasmodesmata (PD) at all cell wall interfaces between the sucrose-producing mesophyll cells and the phloem sieve elements. These PD were shown to be functional in experiments using fluorescent tracer molecules indicating that sucrose diffuses freely between the cells (Turgeon and Hepler, 1989; Liesche and Schulz, 2012a). The higher sugar concentration that has been measured in the phloem compared to the rest of the leaf is explained according to the polymer-trap hypothesis by the conversion of sucrose that enters the specialized phloem companion cells, called intermediary cells (IC), into sugar oligomers (Haritatos et al., 1996; McCaskill and Turgeon, 2007). As the sucrose concentration is thereby constantly reduced, it can be replenished by diffusion from mesophyll to phloem along the sucrose concentration gradient. The sugar polymers are deemed too large to pass through PD on the bundle sheath cell (BSC) side, but can pass on into the sieve element to be transported in the phloem.

Circumstantial evidence for this theory is given by the IC-specific expression of sugar-oligomerizing enzymes (Beebe and Turgeon, 1992; Haritatos et al., 2000; Volk et al., 2003) and the abundance of PD with a unique structure at the BSC-IC interface. These PD are highly branched, with more branches on the IC side (Turgeon et al., 1975; Volk et al., 1996). These branches on the IC side are generally described as very narrow, with no cytoplasmic sleeve visible between the plasma membrane and the desmotuble (Fisher, 1986; Turgeon et al., 1993). The only indication that the PD can indeed fulfill a filtering function as anticipated in the polymer-trap hypothesis comes from expression of raffinose biosynthesis genes in tobacco companion cells, which does not result in efficient phloem transport, probably because they lack the structural specialization of IC (Hannah et al., 2006).

It is generally assumed that small molecules (below 1 kD) can freely diffuse from cell to cell through a cytoplasmic sleeve in the PD between plasma membrane and desmotuble (Terry and Robards, 1987; Maule et al., 2011). Only in one case has selective transport of small molecules been shown: small fluorescent tracers could pass from a leaf epidermal cell into the basal cell of trichomes, but not the other way round (Christensen et al., 2009). This phenomenon remains unexplained. Size-dependent filtering by PD is well-established for larger molecules, like proteins, and PD in different tissue were found to have different size-exclusion limit (Kim et al., 2005).

In the present work, we test the validity of the polymer-trap hypothesis by theoretical modeling of diffusion of sucrose, raffinose, and stachyose through PD with different substructural configurations. Potential configurations are predicted by comparing the model data with the experimentally determined diffusion coefficient at the interface in question. The predictions are evaluated using literature data on sugar concentration potentials and flow.

## Theory and results

### Hydrodynamic radii of sucrose, raffinose, and stachyose

The size of the molecules in question is an essential parameter for the following considerations on plasmodesmal filtering. The size of the hydrated molecule is reflected in the hydrodynamic radius. Hydrodynamic radii can be calculated using the Stokes–Einstein equation after determining the diffusion coefficient experimentally (Pappenheimer, 1953; Schultz and Solomon, 1961).

(1) $$R_{\text{hyd}}=\frac{KT}{D6\pi \eta }\times 10^{17}$$

with Boltzman's constant $K(1.38\times 10^{-23}\;\frac{J}{K})$, absolute temperature *T* (298 K), diffusion coefficient of the sugar *D*, and viscosity η (0.89 centipoise for water). Values for sucrose, raffinose, and stachyose as well as the symplasmic tracer fluorescein derived in this way are listed in Table 1.

**Table 1:**
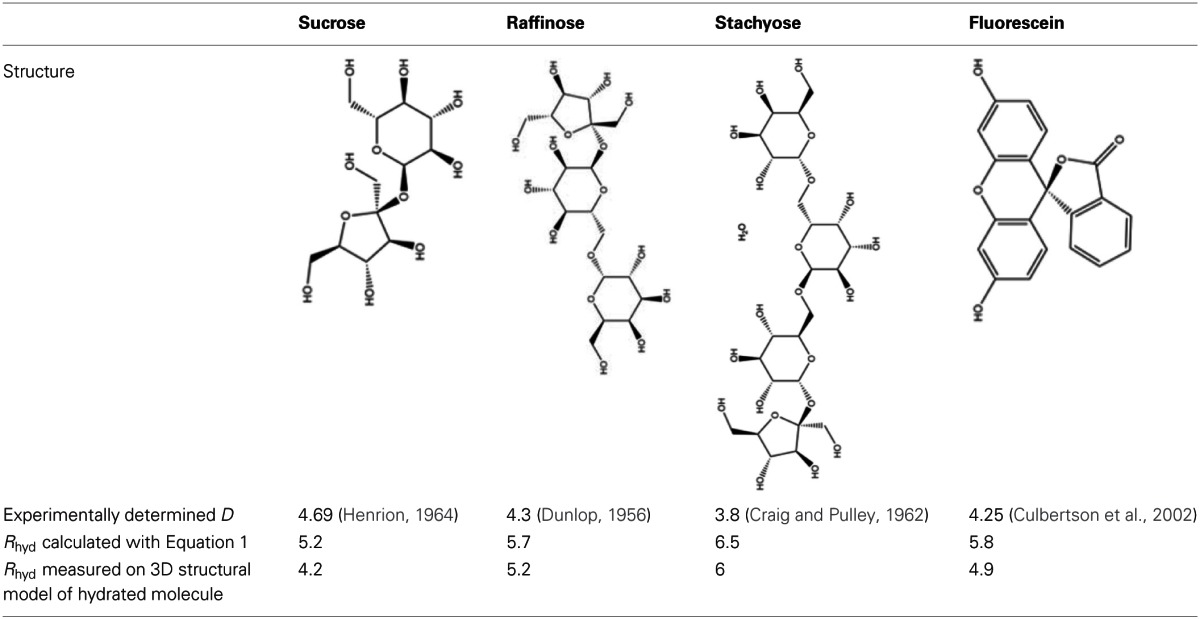
**Diffusion coefficients *D* and hydrodynamic radii *R*_hyd_ of relevant sugars and the fluorescent tracer fluorescein**.

*All D were determined in water at 25 °C. R*_hyd_ *determined from 3D structure of hydrated molecules was measured as the shortest radius in order to take the cylindrical form of molecules into account.*

The Stokes–Einstein equation returns the radius of a sphere that diffuses at the same rate as the molecule in question. This might influence our considerations on filtering as the sugar molecules are not spherical but cylindrical in form. Therefore, measurement of hydrodynamic radius was performed on models of the hydrated molecules in the software ChemOffice3D. The shortest model radii of the three sugars are shorter than the calculated ones because of their cylindrical form, which results in a smaller difference between raffinose and stachyose (Table 1). Even though the different approaches yield slightly different hydrodynamic radii, the relative values are very similar, especially for the difference between sucrose and raffinose, which is decisive in our considerations. In the following, values for hydrodynamic radius derived from the molecular model are used as they closer reflect the actual dimensions of hydrated sugars.

### The substructure of plasmodesmata

The ultrastructural analysis of PD has, so far, been limited to approaches using transmission electron microscopy (TEM; Roberts and Oparka, 2003). With optimal tissue preparation and imaging, structures inside PD were resolved to the extend that models of PD substructure could be proposed (e.g., Ding et al., 1992; Waigmann et al., 1997; Botha et al., 1993). Whereas all of the proposed models agree on the presence of a central desmotuble and plasma membrane lining the outer PD wall, they differ in the arrangement of particles that restrict the cytoplasm between desmotuble and plasma membrane. Freeze-substituted PD of developing tobacco leaves showed electron-dense particles attached to the desmotuble as well as to the outside of the plasma membrane in the neck region (Ding et al., 1992). Image overlays indicate that these particles restrict the cytoplasmic sleeve to channels of ~12.5 Å in radius. The central part of PD was found to be wider and spoke-like extensions were present between desmotuble and plasma membrane (Ding et al., 1992). Both features were also shown for the central part of conventionally fixed PD from pea roots (Schulz, 1995).

The neck region of tobacco leaf trichome PD differs from the mesophyll PD in that they do not have electron-dense particles attached to desmotuble and plasma membrane (Waigmann et al., 1997). Earlier experiments demonstrated a larger size-exclusion limit of the trichome PD compared to mesophyll PD (Waigmann and Zambryski, 1995), which might be enabled by the unobstructed sleeve structure (Waigmann et al., 1997). Extensive measurements of the neck region of PD in pea roots on 30 nm ultrathin sections revealed a half-sleeve width of 16 ± 0.18 Å for controls and different mannitol treatments (*n* = 205; Schulz, 1995).

The models with nano channels and cytoplasmic sleeve dominate the discussion of PD structure today, but alternative configurations are possible, as there are many structures that are not dense enough to be picked up by TEM. In nuclear pores, a hydrogel structure was shown to be instrumental to selective transport through the pore (Miao and Schulten, 2009). These hydrogel parts are not visible on TEM images (Panté, 2007; Maimon et al., 2012). In PD, no analogs to the flexible filamentous phenylalanine-glycine nucleopore proteins (FG-Nups) have been identified to date. These intrinsically disordered proteins form the backbone of the hydrogel in nuclear pores (Frey and Görlich, 2007; Terry and Wente, 2009; Mincer and Simon, 2011). However, in general, only relatively few PD proteins are known (Fernandez-Calvino et al., 2011), and approaches might not have been suitable to identify long-chained membrane-attached proteins. A hydrogel filling the cytoplasm in PD could potentially enhance their filtering capacity, as hydrogels act as molecular sieves (Amsden, 1998a; Dembczynski and Jankowski, 2000; Zhang and Amsden, 2006).

**Figure 1:**
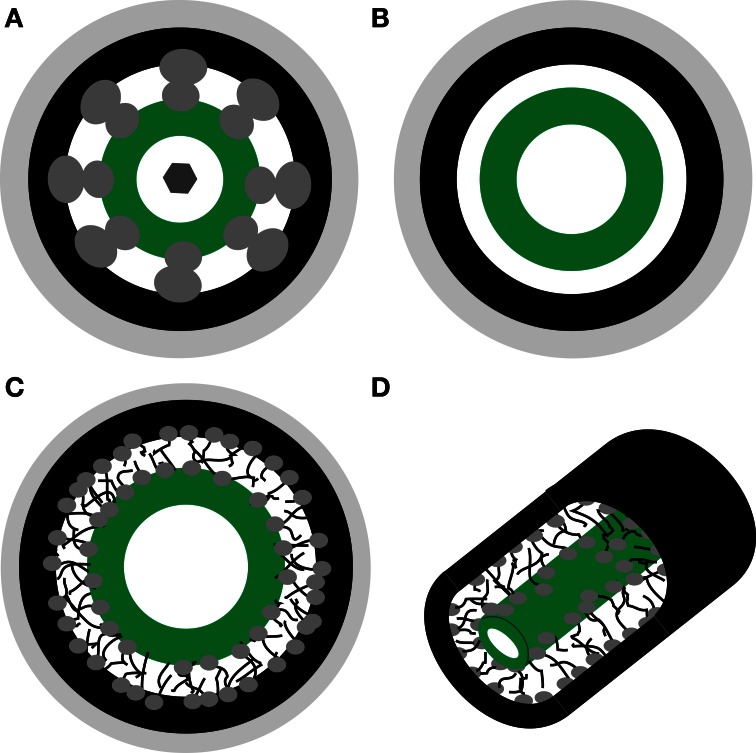
**Schematic representations of hypothetical plasmodesmata substructure. (A)** Nano channel model proposed by Ding et al. (1992). **(B)** Cytoplasmic sleeve model proposed by Waigmann et al. (1997). **(C,D)** Cytoplasmic sleeve filled by polymer-meshwork hydrogel. Light gray—cell wall, black—plasma membrane, dark gray—proteins, green—desmotuble.

The structure of PD at the BSC-IC interface is unlike that of any other PD with the long narrow neck regions on the IC side appearing filled by the desmotuble on TEM images (Fisher, 1986; Turgeon et al., 1993; Volk et al., 1996). Small openings, below 1 nm, between PM and desmotuble are not expected to be resolved by TEM. Based on the PD models described above, we include three hypothetical configurations in the subsequent analysis of diffusional properties (Figure 1).

### Modeling diffusion hindrance in plasmodesmata with different substructure

Diffusion through a pore is hydrodynamically constrained when the dimensions of a solute molecule are of the same order as those of the pore. To describe diffusion in this case, the hindrance factor *H* has to be included in Fick's law of diffusion:

(2) $$J=HD_{0}\frac{△c}{d}$$

with the flux per unit area *J* in mol μm^−2^ s^−1^, diffusion coefficient in bulk solution *D*_0_ in μm^2^ s^−1^, concentration potential delta *c* in mol μm^−3^ and the diffusion distance d in μm. The hindrance factor describes the relation of the apparent diffusion coefficient *D*_a_ to the diffusion coefficient in bulk solution *D*_0_.

(3) $$H=\frac{D_{a}}{D_{0}}$$

Many different models were proposed to describe diffusion under various conditions. In the following, the three most relevant models that describe the potential configurations of PD (Figure 1) are applied to assess relative hindrance of sucrose, raffinose, and stachyose.

#### Sub-nano channels (Figure 1A)

Various models that describe hindered diffusion of molecules through liquid-filled pores are reviewed by Deen (1987) and Dechadilok and Deen (2006). We use the model proposed by Dechadilok and Deen (2006), which is a slightly extended version of the model that was provided by Higdon and Muldowney (1995). The decisive factor is the relative size of solute to pore radius $\lambda =\frac{r_{s}}{r_{p}}$.

(4) $$\begin{array}{l} \text{​​​​}H=1+\frac{9}{8}\lambda \ln\lambda -1.56034\lambda +0.528155\lambda^{2}+1.91521\lambda^{3}\\ \;-\;2.81903\lambda^{4}+0.270788\lambda^{5}+1.10115\lambda^{6}-0.435933\lambda^{7} \end{array}$$

This model accounts for the effects of particle-wall hydrodynamic interactions and steric restrictions on diffusion of a neutral particle through pores that are several times longer than wide. The model covers a wide range of λ.

**Figure 2:**
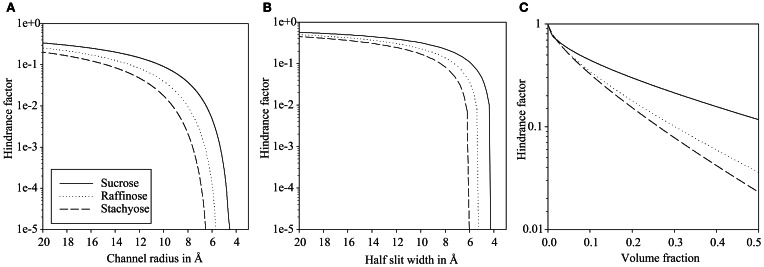
**Logarithmic scale plots of diffusion hindrance in relation to hypothetical plasmodesmata substructure configurations.** Hindrance factor for sucrose, raffinose, and stachyose increases faster with smaller dimensions in single plasmodesmal channels **(A)** than in a cytoplasmic sleeve **(B)**. Hindrance in hydrogels **(C)** is moderate, even at high volume fractions.

The hindrance factor for sucrose, raffinose, and stachyose in dependence of the channel radius are given in Figure 2A. At 20 Å channel radius the apparent diffusion of sucrose is reduced to one third of the value in bulk solution. Diffusion of raffinose and stachyose is only slightly more hindered compared to bulk solution with 20 and 26% respectively. A 1000-fold reduction of diffusion is realized at 5.4 Å for sucrose, 6.5 Å for raffinose, and 7.6 Å for stachyose, i.e., close to the molecular size cut-off. At the stachyose cut-off of 6.1 Å, raffinose diffusion is hindered 60 times more than sucrose.

The results are virtually identical to values calculated with the model provided by Mavrovouniotis and Brenner (1988), which is specifically developed for cases where the channel radius *r*_*c*_ is only slightly larger than the radius *r*_*s*_ of the diffusing solute (data not shown).

#### Slit (Figure 1B)

The cytoplasmic sleeve forms a ring between PM and desmotuble with very small width compared to the ring diameter and can therefore be described as a slit. An analytical solution to describe diffusion through slits was provided by Dechadilok and Deen (2006). Lambda is here the relative size of the solute molecule to half slit width *h*:$\lambda =\frac{r_{s}}{h}$.

(5) $$\begin{array}{l} H_{\text{slit}}=1+\frac{9}{16}\lambda \ln\lambda -1.19358\lambda +0.4285\lambda^{3}\\ \;-\;0.3192\lambda^{4}+0.08428\lambda^{5} \end{array}$$

As shown in Figure 2B, diffusion hindrance is moderate for a slit of 20 Å half-width with reduction to 57% for sucrose, to 50% for raffinose, and to 45% for stachyose diffusion. A 1000-fold reduction of diffusivity is first realized at half-width of 4.4, 5.4, and 6.2 Å for sucrose, raffinose, and stachyose, respectively. At the cut-off of stachyose of 6.1 Å, raffinose diffusion is hindered three times more than sucrose.

Compared to the sub-nano channels, the relative hindrance of raffinose and stachyose in relation to hindrance of sucrose is lower at larger radii/slit width, but close to the molecular cut-off, relative hindrance is an order of magnitude higher than in the channel configuration (Figure 2B).

#### Hydrogel (Figures 1C,D)

Models that describe hindered diffusion through hydrogels are reviewed by Amsden (1998b) and Waters and Frank (2009). The parameters that influence hindrance are the polymer volume fraction and polymer fiber radius. We use the model described by Philips, as it combines terms for hydrodynamic interactions and obstruction effects and was shown to fit experimental data over a wide range of parameter values (Phillips, 2000).

(6) $$H=\exp{\left(-0.84\right)}^{1.09}\times \exp{\left(-\left(3.727-2.46\lambda +0.822\lambda^{2}\right)\phi \right)}^{\left(0.358+0.366\lambda -0.0939\lambda^{2}\right)}$$

Term explanation: ratio of fiber radius to solute radius $\lambda =\frac{r_{f}}{r_{s}}$, adjusted volume fraction $f={\left(1+\frac{r_{s}}{r_{f}}\right)}^{2}\Phi$, Φ is actual polymer volume fraction. We used a polymer fiber radius *r*_*f*_ of 15 Å for calculations, anticipating protein filaments, similar to the ones found in the nuclear pore outer area (Mincer and Simon, 2011).

Hindrance of such a hydrogel for small molecules, like the sugars in question here, is moderate (Figure 2C). Even at extremely high volume fractions, sucrose diffusion is reduced by less than 10 times and raffinose and stachyose less than 50 times. A volume fraction of 0.5 actually means that half the gel volume is filled with polymers. Typical volume fractions of hydrogels are below 0.2 (Amsden, 1998b), the FG-repeats in the nuclear pore make up between 0.12 and 0.2 of the pore volume (Frey and Görlich, 2007).

The calculations only apply to a pure hydrogel. In PD, it could be expected that a hydrogel would fill up the space within a sub-nano channel or sleeve. The hindrance effects of the gel would then be increased by wall effects, similar to the ones described in the previous sections. Since no information is available on the exact geometries of either the cytosolic cross-section in the PD neck region or the hydrogel properties, we do not attempt to accommodate the combined effect of channel/slit- and hydrogel diffusion hindrance in our considerations.

When half the gel space is taken up by polymers, raffinose diffusion is hindered 3.2 times more than sucrose. Relative hindrance of raffinose and stachyose in relation to hindrance of sucrose is, even at extremely high volume fractions, several orders of magnitude lower than relative hindrance of sub-nano channel and slit configurations with low radius/slit width.

It can be concluded that efficient filtering is only possible in sub-nano channels and slit when the channel radius or half slit width is very close to the hydrodynamic radius of raffinose and stachyose. Hydrogels, even at very high polymer volume fraction, show only a modest relative hindrance.

### Theoretical data matches the experimentally determined diffusion coefficient

In order to test if the theoretical data on hindrance in the three hypothetical configurations is physiologically relevant, it was compared to the experimentally determined diffusion coefficient of the PD at this specific interface. The effective diffusion coefficient for the fluorescent tracer molecule fluorescein was determined with live-cell photoactivation microscopy as described in Appendix. Fluorescein has a hydrodynamic radius of 4.9 Å, as determined with ChemOffice Pro, meaning that its size lies between sucrose and raffinose (Table 1).

Determination of the apparent diffusion coefficient across the cell wall between IC and BSC in *Cucurbita pepo* with photoactivation microscopy yields a value of 0.41 μm^2^ s^−1^ for fluorescein. The value is actually very close to the diffusion coefficient across the cell wall between mesophyll cells, which is around 0.5 μm^2^ s^−1^ assuming a cell wall thickness of 0.1 μm (Liesche and Schulz, 2012b). Compared to diffusion in water, these values are about 10 times lower.

The effective diffusion coefficient *D*_eff_ per PD is calculated from the hindrance factors calculated above by,

(7) $$D_{\text{eff}}=x(H\times D_{\text{cyt}})$$

with *x* the number of PD per μm^2^ cell wall interface, *H* the hindrance factor and *D*_cyt_ the cytosolic diffusion coefficient. The cytosolic diffusion coefficient can be assumed to be two times lower than that in water, based on measurements using electron spin resonance (Mastro et al., 1984) or fluorescent tracers (Liesche, unpublished data). In case of sub-nano channels, the value has to be additionally multiplied by the number of channels per PD. Volk et al. (1996) calculated a PD frequency on the IC-side of *Cucumis melo* of 14 PD per μm^2^. A similar value can be estimated from *Cucurbita pepo* micrographs (Turgeon et al., 1975). The value refers to PD on the IC-side, which are, due to their narrower diameter, likely to be transport-limiting.

**Figure 3:**
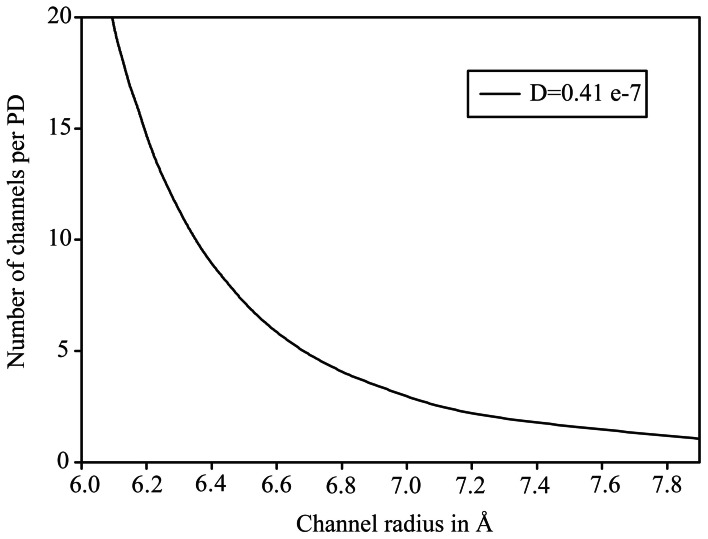
**Plot of effective diffusion coefficient for diffusion of fluorescein through plasmodesmata with sub-nano channel configuration.** The curve shows at which combination of number of channels and channel radius the experimentally determined value is matched.

We chose the experimental value of 0.41 μm^2^ s^−1^ to find PD configurations where the theoretical model would yield a matching diffusion coefficient. With 14 PD per μm^2^, a slit would need to have a half width of 5.2 Å to match the effective diffusion coefficient, whereas a hydrogel even at just below 0.5 volume fraction would match this value. Results for the sub-nano channel configuration are plotted in Figure 3 to show which combination of channel radius and number of sub-nano channels per PD, the experimental value is met. At nine channels, suggested by Terry and Robards (1987) for *Abutilon* nectary trichome cells, each channel would need to have a radius of 6.4 Å.

The extremely restricted diffusion as considered in the theoretical models does fit experimental data. Since the effective diffusion coefficient is quite low, it is met even when hindrance is very high, because of the high number of PD at this interface.

### Testing filtering efficiency and physiological relevance of the modeled PD configurations with literature values

Using literature values for sugar concentration potentials between IC and BSC, we can test if the model configurations at which the effective diffusion coefficient *D*_eff_ matches experimental data would enable efficient filtering. In order to test whether the model parameters are feasible, we have tested the models with literature values of sucrose flux into the phloem.

Haritatos et al. (1996) provided values for the concentration of different sugars in the sieve element-IC complex and leaf mesophyll in *Cucumis melo*. The concentration of sugars in the IC is generally assumed to be similar to that in sieve elements as they are connected via wide PD. Similarly, the concentration in BSC can be assumed to be similar to the rest of the mesophyll cells, because of their high cytosolic coupling (Liesche and Schulz, 2012a). The authors found the concentration of raffinose and stachyose in mesophyll cells to be virtually 0 (Haritatos et al., 1996). Based on the concentration values, a potential across the BSC-IC interface of 67 mM for raffinose and 334 mM for stachyose can be calculated. The sucrose concentration potential in the cytosol between mesophyll and IC in *Cucumis melo* was determined as 60 mM (Haritatos et al., 1996).

The only values for vein loading rates in mature leaves of an active symplasmic loader that we are aware of are provided by Schmitz et al. (1987) for *Cucumis melo*. Leaf export rate as determined with ^14^CO_2_ labeling experiments was 4.25 mg (CH_2_O) per dm^2^ of leaf per h. Since the BSC-IC interface area in 1 dm^2^ of leaf is given as 3558 mm^2^, the flux across this interface was calculated to be 1.1 × 10^−5^ (CH_2_O) or 9.7 × 10^−7^ mol sucrose m^−2^ s^−1^mol sucrose. This value is in the range of comparable values reported for flux across the BSC-companion cell interface in active apoplasmic and passive symplasmic loading species (Table 2). The value is furthermore one order of magnitude lower than flux out of sieve elements in phloem unloading in the root tip of peas, which is entirely symplasmic (Schulz, 1998).

**Table 2:** **Sucrose flux rates across the bundle sheath-companion cell interface reported in the literature**.

**Flux in mol sucrose m^−2^ s^−1^**	**Species**	**Transport type**	**Source**
1.3 × 10^−7^	*Fagus sylvatica*	Symplasmic	Münch, 1930
1.6 × 10^−7^	*Beta vulgaris*	Apoplasmic	Fondy and Geiger, 1977
3.3 × 10^−7^	*Nicotiana tabacum*	Apoplasmic	Cataldo, 1974
8.2 × 10^−7^	*Beta vulgaris*	Apoplasmic	Sovonick et al., 1974
9.7 × 10^−7^	*Cucumis melo*	Symplasmic	Schmitz et al., 1987
2.3 × 10^−6^	*Triticum aestivum*	Apoplasmic	Kuo et al., 1974
2.9 × 10^−6^	*Pisum sativum*	Apoplasmic	Wimmers and Turgeon, 1991
6.9 × 10^−5^	*Pisum sativum*	Symplasmic (unloading)	Schulz, 1998

Sugar flux using the hindrance factors that were determined for the different hypothetical PD configurations is calculated with Equation 2. Rearrangement of Equation 2 to,

(8) $$△c=\frac{J}{HD_{0}}d$$

allows for calculation of sugar concentration potential. A diffusion distance of *d* = 0.18 μm corresponding to the length of the neck region, as measured by Fisher (1986) in *Coleus blumei*, was assumed. The rest of the PD is considerably wider and therefore not likely to be transport-limiting.

**Figure 4:**
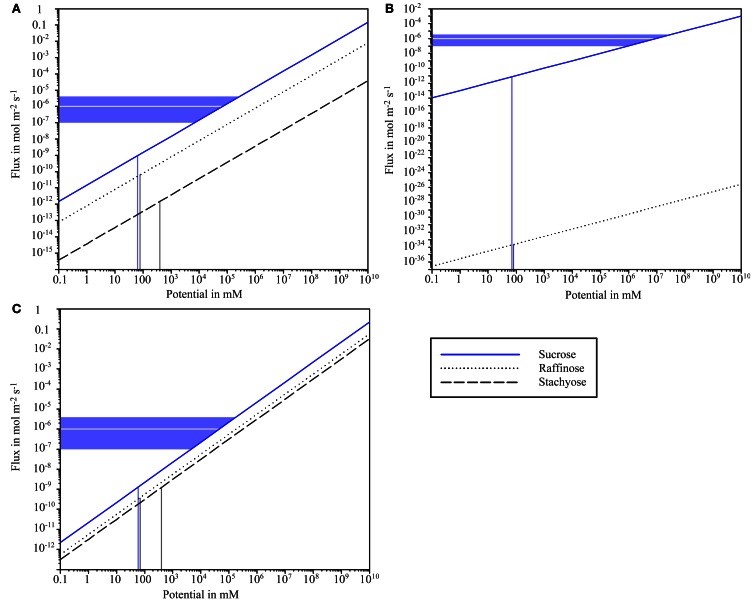
**Logarithmic scale plot of sugar flux and concentration potential between bundle sheath cells and intermediary cells for different plasmodesmata configurations. (A)** Sub-nano channel configuration assuming 9 channels with 6.5 Å radius. **(B)** Slit configuration assuming half slit width of 5.2 Å. **(C)** Hydrogel configuration assuming a polymer volume fraction of 0.49. Vertical lines indicate sugar concentration potentials as provided by Haritatos et al. (1996). Horizontal lines indicate sucrose flux values provided by Schmitz et al. (1987, gray) and a flux range based on values listed in Table 2 (blue). Raffinose flux is strongly hindered in the slit model in contrast to the other configurations, which would enable considerable “leakage” out of the intermediary cell. Hindrance of sucrose flux at concentration potentials observed by Haritatos et al. (1996) would in all models be orders of magnitude too high to realize flux rates reported by Schmitz et al. (1987) and other authors.

Assuming a sub-nano channel configuration with 9 channels of 6.4 Å radius, the literature values for sugar concentration potential would lead to flux across the BSE-IC interface of 58 × 10^−12^ mol m^−2^ s^−1^ and 1.2 × 10^−12^ mol m^−2^ s^−1^ for raffinose and stachyose, respectively, while allowing a sucrose flux of 860 × 10^−12^ mol m^−2^ s^−1^ (Figure 4A). The sucrose flux values from *Cucumis melo* (Schmitz et al., 1987) would be met by sub-nano channels at a potential of ~10^5^ mM. The results are almost identical for sub-nano channel configurations with different combinations of channel number and radius as determined in Figure 3 (data not shown).

Assuming a slit configuration with 5.2 Å half-slit width, the concentration potentials given by Haritatos et al. (1996) would lead to an extremely low flux of raffinose with 2.4 × 10^−34^ mol m^−2^ s^−1^, while stachyose would be completely blocked (Figure 4B). Sucrose would flow into IC at a rate of 6.2 × 10^−12^ mmol m^−2^ s^−1^, which is several dimensions lower than flux across this interface as stated in the literature. The sucrose flux values provided for *Cucumis melo* (Schmitz et al., 1987) and the values calculated for other plant species (Table 2) would require an unrealistic potential of around 5 × 10^7^ mM (Figure 4B).

Hydrogel-filled PD channels with a polymer volume fraction of 0.49 would enable flux of 0.4 × 10^−9^ mmol m^−2^ s^−1^ and 10^−9^ mol m^−2^ s^−1^ for raffinose and stachyose, respectively at the concentration potentials provided by Haritatos et al. (1996) (Figure 4C). The sucrose flux values from *Cucumis melo* (Schmitz et al., 1987) would be met by the hydrogel at a potential of around 5 × 10^4^ mM (Figure 4C).

The calculations show the slit configuration as significantly more efficient filter for raffinose and stachyose than the other configurations. Conductance (flux/potential) is very similar for the sub-nano channel configuration and the hydrogel (Figures 4A,C). The slit model enables significantly less flux than the other configurations at a comparable potential (Figure 4B). Flux values are obviously far below the value that would be required to drive the observed flow through the theoretical PD configurations assumed here.

## Discussion

The results of this study provide insight on four key questions regarding the feasibility of sugar filtering in active symplasmic phloem loading:

### Can plasmodesmata discriminate small differences in hydrodynamic radius?

The theoretical considerations of diffusion hindrance through PD with hypothetical substructural configurations show that efficient filtering of raffinose and stachyose is feasible when the channel radius or slit width is very close to the physical size of the transportate. In principle this could be the case at the cell wall interface between BSC and IC in RFO-transporting plants. The relatively low diffusion coefficient for symplasmic transport across the BSC-IC interface in *Cucurbita pepo* that we determined experimentally, confirms that PD indeed have a very limited permeability. This can be explained by a restricted cross-sectional area per PD available for flow. The diffusion coefficient is a value integrated over all PD present at this crucial interface. One could argue that the observed values could also be due to a small number of relatively wide PD that enable the observed diffusion, while most of the very abundant PD in this interface would be completely blocked. We consider this alternative interpretation as very improbable. Accordingly, the experimental data support the hypothesis that PD at the BSC-IC interface are specialized and have a very small passage area for cytosolic compounds. For the sub-nano channel configuration we find a raffinose flux rate reduced to 6.7% that of sucrose. Values in this range were proposed to allow efficient RFO trapping according to Haritatos and Turgeon (1995). The authors calculated a raffinose permeation factor of 1.9% that of sucrose in PD pores of 7 Å radius. The relative permeation is not derived from flux rates but from a geometrical pore size factor. Nevertheless, this pore size factor is in principle comparable to our hindrance factors as it introduces the same effects of steric hindrance and wall interaction (although using different equations) into the diffusion equation.

### Is diffusion the sole mechanism of pre-phloem transport in active symplasmic loaders?

Although our modeling supports the possibility that PD in the BSC-IC interface fulfill the filtering function that has been ascribed to them as part of the polymer trap mechanism (Turgeon et al., 1993), comparison to literature values for sucrose flux into the IC does not seem to support this conclusion. The concentration potential that would be necessary to realize the flux into IC observed in *Cucumis melo* (Schmitz et al., 1987) would need to be orders of magnitude higher than what is physiologically feasible, especially in the slit configuration that would offer the best filtering efficiency. Even for the sub-nano channel model a potential of 10^5^ mM would be needed to enable sufficient diffusional sucrose flux (see Figure 4A). This value is far higher than any sugar concentration potential between adjacent plant cells reported so far and about 1600 times higher than what has been calculated for the BSC-IC interface in *Cucumis melo* (Haritatos et al., 1996).

It should be noted that the methodology could have influence on the discrepancy between the values for diffusion coefficient and sucrose flux. In contrast to the sugar molecules, the fluorescent tracer used here carries a negative charge, potentially altering intra-PD diffusion kinetics. While it is generally assumed that only the hydrodynamic radius influences passage through PD (Terry and Robards, 1987), one study reported minimally reduced diffusion of charged GFP compared to neutral GFP (Dashevskaya et al., 2008). Nevertheless, it is extremely unlikely that sucrose diffuses thousands of times faster than a charged molecule of the same hydrodynamic radius, which would be necessary to reconcile diffusion and flux values.

Another factor that influences the calculations is the length of restricting part of the PD (*d* in Equation 8). If it was half as long as assumed here, flux would be twice as high. However, electron micrographs have shown the whole part of the PD on the IC side to be “narrow” and no special protein aggregations have been reported that could suggest more local restrictions.

How can the fact that diffusion driven by a concentration potential is insufficient to realize the observed sucrose flux rates be explained? Wider PD could be possible if the filtering would not depend on size-exclusion based on the hydrodynamic radius, but were realized instead by a gating mechanism. Such a mechanism is implied in the case that PD transport compounds unidirectional, as has been demonstrated for tobacco leaf trichomes (Christensen et al., 2009). A solely unidirectional mechanism can, however, be excluded here according to preliminary experiments with uncaging of fluorescent tracers that were passing the BSC-IC interface in both directions (Liesche, unpublished data).

RFO concentrations in the BSC used in the present study to determine the concentration potentials are very small which is taken as argument for efficient filtering. However, the capacity for raffinose and stachyose breakdown in BSC would allow wider, i.e., less restrictive PD. Alpha-galactosidase activity has been demonstrated in the leaves of the *Cucurbitaceae*, although strongly declining after sink-source transition (Pharr and Sox, 1984; Gaudreault and Webb, 1986). If enzymatic breakdown could happen fast enough in the BSC, it could compensate for RFO “leakage” due to less efficient filtering. This should be tested, for example by BSC-specific gene expression analysis and *in vitro* activity assays.

In conclusion, the transport of sucrose from mesophyll into IC cannot solely be driven by diffusion, though particular adaptations of the PD substructure, such as a gating mechanism or filling of the sub-nano channels with hydrogel, and the enzymatic activity in BSC would reduce the discrepancy between flux rate and the postulated differences in concentration potential of the sugars involved.

### Does apoplasmic sucrose loading into intermediary cells complement symplasmic transport?

For the polymer trap hypothesis it is important that the narrow PD allow sucrose to enter the IC with a rate sufficient to match the phloem export of RFO sugars. It could be argued that insufficient symplasmic sucrose transport is complemented by apoplasmic sucrose uptake into IC, and that the main function of the specific PD is to restrict leakage of RFO back into the BSC. AP-chloromercuribenzene-sulfonic acid (PCMBS), an inhibitor of sugar transporters was shown to significantly reduce phloem loading in *Cucurbita pepo* using C-11 (Thorpe and Minchin, 1988). However, this observation, and thus the possibility for additional apoplasmic sucrose uptake is contradicted by a number of thorough PCMBS studies on active symplasmic loaders which do not show a reduction of phloem loading (cf. Weisberg et al., 1988; van Bel et al., 1993; Turgeon and Medville, 2004), and by the downregulation of sucrose transporters which had almost no effect on phloem loading (Zhang and Turgeon, 2009). Moreover, PCMBS does not only block sugar transporters, but also aquaporins that are important for the osmotic uptake of water into the SECCC (Heinen et al., 2009). Obviously, this possible side effect has to be considered in the interpretation of PCMBS data.

### Can the reported flux rates across the BSC-IC interface be explained with bulk-flow?

As alternative to diffusion, it has been hypothesized that sucrose enters IC by bulk flow. Voitsekhovskaya et al. (2006), who could not detect a cytosolic sucrose concentration potential along the pre-phloem pathway of the symplastic loader *Alonsoa merionalis*, speculated that the negative water potential in the phloem draws water from the bundle sheath. This could create a mass flow that moves sucrose into the phloem, while preventing diffusion toward the mesophyll. A number of factors have influence on the hydrostatic pressure of each of the cells on the pre-phloem transport pathway, such as cytosolic and vacuolar sugar concentration, conversion into transitional starch in chloroplasts, water potential in the apoplast and its domains, frequency, and distribution of aquaporins, extensibility of the cell wall and last not least the frequency and permeability of PD. We can assume a well-regulated sucrose homeostasis in these cells with set values that balance concentration potentials with osmotic water uptake not only in active, but even more so in passive symplasmic loaders, such as gymnosperm trees (see Liesche et al., 2011; Liesche and Schulz, 2012a). Bulk flow from mesophyll into the sieve elements is as well-postulated for passive symplasmic loading angiosperm trees (Turgeon, 2010; Fu et al., 2011). It is interesting to note that, irrespective of the loading mode, sucrose flux rates across the BSC-IC interface, respectively that between BSC and companion cell-sieve element complex are very similar (Table 2).

With regard to active symplasmic loaders, we plan to test the bulk flow hypothesis, characterize the minor vein apoplasm with apoplasmic tracers and determine whether the PD-mediated cell wall permeability of the BSC-IC interface is significantly higher for the transport into the IC than from IC to BSC. This would indicate that flow is not simply diffusive but somehow favored toward the IC direction.

The results show that important questions regarding the mechanism of active symplasmic phloem loading remain unsolved. There is no doubt that the distinctive PD at the BSC-IC interface play a significant role in phloem loading. The experimentally determined comparatively low diffusion coefficient across the BSC-IC interface together with the high PD abundance shown in all TEM investigations of active symplasmic loaders is consistent with the interpretation that they are bottle necks in symplasmic transport, and that tri- and tetrasaccharides experience a significantly larger hindrance through these PD than sucrose. The data provided here indicates that additional factors are yet to be discovered that enable efficient sugar filtering while also allowing sufficiently high sucrose transport rates.

### Conflict of interest statement

The authors declare that the research was conducted in the absence of any commercial or financial relationships that could be construed as a potential conflict of interest.

## Figures and Tables

**Figure A1:**
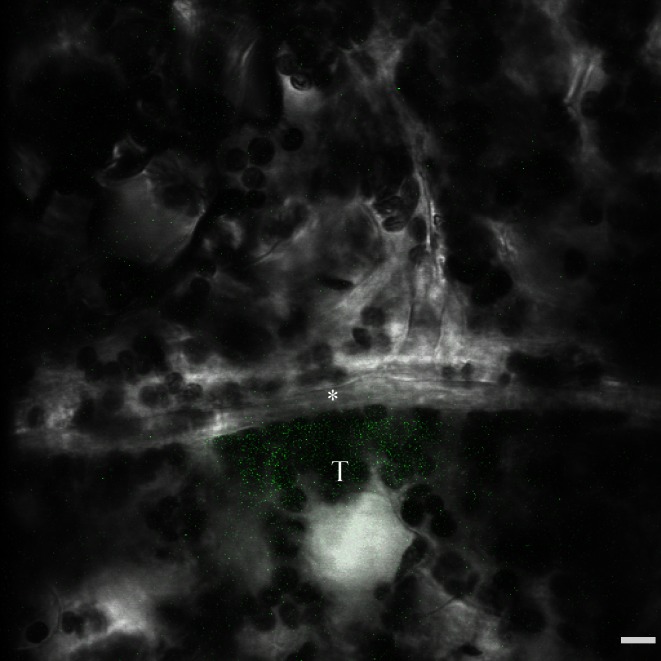
**Confocal image of a photoactivation experiment in a *Cucurbita pepo* leaf.** The image shows a single optical section of a 30 μm thick z-stack that was recorded at one time point during the 10 s-long photoactivation phase. The fluorescence of the activated tracer is displayed as extended focus projection in green, overlaid over the brightfield image. In image analysis, three-dimensional regions of interest are drawn adjacent to the cell wall between the bundle sheath target cell (T) and the intermediary cell (*) in order to quantify diffusion of activated tracer. Scale bar: 10 μm.

## Appendix

### Experimental determination of the diffusion coefficient of fluorescein at the intermediary cell-bundle sheath cell wall interface

The experimental procedure to determine the diffusion coefficient at the BSC-IC interface in the active symplasmic loader *Cucurbita pepo* was very similar to the quantification of PD-mediate cell wall permeability between *Cucurbita maxima* leaf mesophyll cells as described by Liesche and Schulz (2012b). Briefly, the photoactivatable tracer caged fluorescein (Invitrogen, USA) is applied to the target tissue, where it is taken up passively into all cells. Installing the tissue, in this case a whole leaf still attached to the plant, on a microscope, the tracer can then be activated by UV illumination in a specific target cell and its spread to neighboring cells monitored (Figure A1). The use of a confocal microscope equipped with resonant scanner (SP5, Leica Microsystems, Germany) allows for collection of three-dimensional data with high signal-to-noise ratio combined with high speed acquisition. From 3D time series all necessary functional and anatomical data for each individual cell can be gathered. The effective diffusion coefficient is calculated with Fick's law of diffusion.

(9) $$J=D_{\text{eff}}\frac{△c}{d}$$

The effective diffusion coefficient was determined to be 4.087e-7, standard deviation 2.878e-7, standard error 1.439e-7, *n* = 4.

As experiments were performed, it could not be discriminated between IC and phloem parenchyma cells, which can have a similar shape as IC and are also located adjacent to BSC (see Figure 1A in Turgeon and Hepler, 1989). Instead, this was done during image processing, based on the very different ratio of cellular to vacuolar volume of the two cell types. Two populations corresponding to the two cell types were identified: one with the cytosol making up 31–56% of the cell volume, the other with less than 10% cytosol. Experiments that involved the latter cells were not considered as they are likely to be phloem parenchyma cells that are known to have a much larger vacuole than IC (Turgeon et al., 1975). A mix-up with companion cells that can be present in on the adaxial side of veins of *C. pepo* (Turgeon et al., 1975) can be excluded as imaging happened from the abaxial side and penetration depths was limited to 150–200 μm.
